# P19 Antiphospholipid antibodies in the context of a varicella zoster infection

**DOI:** 10.1093/rap/rkae117.050

**Published:** 2024-11-05

**Authors:** Stephanie Gall, Nicola Goodson

**Affiliations:** Aintree University Hospital, Liverpool, United Kingdom; Aintree University Hospital, Liverpool, United Kingdom

## Abstract

**Introduction:**

We present a 40-year-old man who developed a dural venous sinus thrombosis (DVST) and pulmonary embolus (PE) following a primary varicella zoster infection (VZV). He was found to have transiently raised anti beta-2-glycoprotein-1 and anticardiolipin antibodies during admission. There have been few case reports in the literature of transiently raised antiphospholipid antibodies following VZV infection and we discuss the association between antiphospholipid antibodies and infection.

**Case description:**

A 40-year-old immunocompetent man, originally from South India, presented with a three day history of a severe frontotemporal headache, nausea and vomiting. He reported that he and his wife had a 10 day history of pruritic dermatosis with macules, vesicles and crusting with no prior history of chicken pox. He did not report other severe systemic symptoms and at the time of presentation his skin lesions were scabbed over. He had lived in South India, then Dubai up until six months ago. He had not had any recent long-haul flights. He had a computed tomography (CT) head in the emergency department (ED) which showed that appearances were concerning for a dural venous sinus thrombosis (DVST), and he was then recalled for a CT venogram. CT venogram confirmed DVST with a possible venous haemorrhage. Neurology advised a thrombophilia screen (taken prior to starting heparin), CT chest abdomen and pelvis (CT TAP) to look for any evidence of malignancy, split dose low molecular weight heparin (LMWH) and HIV and hepatitis screen. Subsequent CT TAP showed no evidence of malignancy but did show an associated acute pulmonary embolus. HIV and hepatitis screen were negative. Echo showed no evidence of thrombus or patent foramen ovale. Thrombophillia screen showed raised anticardiolipin antibodies of 114.7U/mL and raised anti-beta-2-glycoprotein-1 antibodies of 174.5U/mL, lupus anticoagulant was negative. A rheumatology review was requested to ensure that there was no evidence of connective tissue disease given the raised anti-phospholipid antibodies. ANA and ENA were negative with normal C3 and C4. Clinical history and examination showed no clinical evidence to support a connective tissue disease diagnosis. He was treated with low molecular weight heparin with haematology follow up. Subsequent antiphospholipid antibody testing 12 weeks later showed that anticardiolipin and anti-beta-2-glycoprotein-1 antibody testing was normal.

**Discussion:**

Antiphospholipid antibodies (aPL) have been known to be associated with infections, mainly HIV, hepatitis and Covid-19. There are few case reports that have shown that VZV infection can be associated with transiently raised antiphospholipid antibodies. The exact pathophysiology is not understood.

Most studies related to VZV and aPL have been done in children given the higher prevalence of childhood onset primary VZV infection. Although there is limited literature out there regarding this association, the data have shown that those with raised antiphospholipid antibodies have more thrombotic potential. This also seems to be the case in other infections when there is raised aPL.

The exact mechanisms underlying the development of aPL following VZV infection, or other infections, remain incompletely understood. However, it is hypothesised that molecular mimicry or nonspecific immune activation triggered by the viral infection may lead to the production of antiphospholipid antibodies. Additionally endothelial injury caused by VZV infection may contribute to the prothrombotic state in susceptible individuals.

Given there are few case reports in the literature of VZV associated with raised aPL and thrombus it was important in this case to rule out other causes of thrombus, such as malignancy. On searching for evidence of malignancy, it was interesting that this gentleman was also found to have an acute pulmonary embolus, especially given that he had no symptoms of chest pain, breathlessness and he was not tachycardic. On discovering positive aPL it is important to assess for connective tissue disease as this may be a presentation of secondary anti-phospholipid syndrome (APS), however there was no evidence of connective tissue disease in this patient. This case highlights why it is important to repeat the APS screen 12 weeks apart from the first screen.

**Key learning points:**

aPL antibodies can be triggered by infection and, in these individuals, there is more thrombotic potential. It is important to clinically assess for evidence of thrombus and assess that there is no associated connective tissue disease. This case also highlights the importance of rechecking the aPL screen at least 12 weeks apart before making a diagnosis of anti-phospholipid syndrome.

**Learning objectives:**

• Understanding how infection can cause transiently raised aPL.

• Understanding how VZV can affect aPL and increase thrombotic potential.

**Key learning points:**

• Rechecking aPL at least 12 weeks apart is key. As seen in this case, this man had high levels of anticardiolipin and anti-beta-2-glycoprotein-1 antibodies. On repeated testing these were normal. This case highlights why it is important to have two tests at least 12 weeks apart before diagnosing someone with anti-phospholipid syndrome, as extrinsic factors can transiently raise aPL.

• Infections, including VZV can raise aPL. In someone with positive aPL it is important to consider whether this could be raised because of an infection. Infections such as HIV, hepatitis and Covid-19 have been known to raise aPL.

